# Investigating How People Who Self-harm Evaluate Web-Based Lived Experience Stories: Focus Group Study

**DOI:** 10.2196/43840

**Published:** 2023-01-31

**Authors:** Lizzy Winstone, Becky Mars, Jennifer Ferrar, Paul Moran, Ian Penton-Voak, Lydia Grace, Lucy Biddle

**Affiliations:** 1 Population Health Sciences, University of Bristol Bristol United Kingdom; 2 NIHR Biomedical Research Centre at the University Hospitals Bristol NHS Foundation Trust Bristol United Kingdom; 3 School of Psychological Science, University of Bristol Bristol United Kingdom; 4 Samaritans Surrey United Kingdom

**Keywords:** self-harm, lived experience stories, web-based support, self-help, recovery, focus groups

## Abstract

**Background:**

The positive and negative effects of interacting with web-based content on mental health, and especially self-harm, are well documented. Lived experience stories are one such type of static web-based content, frequently published on health care or third-sector organization websites, as well as social media and blogs, as a form of support for those seeking help via the web.

**Objective:**

This study aimed to increase understanding about how people who self-harm engage with and evaluate web-based lived experience stories.

**Methods:**

Overall, 4 web-based focus groups were conducted with 13 people with recent self-harm experience (aged 16-40 years). In total, 3 example lived experience stories were read aloud to participants, who were then asked to share their reactions to the stories. Participants were also encouraged to reflect on stories previously encountered on the web. Data were analyzed thematically.

**Results:**

Overall, 5 themes were generated: stories of recovery from self-harm and their emotional impact, impact on self-help and help-seeking behaviors, identifying with the narrator, authenticity, and language and stereotyping.

**Conclusions:**

Lived experience stories published on the web can provide a valuable form of support for those experiencing self-harm. They can be motivating and empowering for the reader, and they have the potential to distract readers from urges to self-harm. However, these effects may be moderated by age, and narratives of recovery may demoralize older readers. Our findings have implications for organizations publishing lived experience content and for community guidelines and moderators of web-based forums in which users share their stories. These include the need to consider the narrator’s age and the relatability and authenticity of their journey and the need to avoid using stigmatizing language.

## Introduction

### Background

Self-harm is an important public health priority, with increased prevalence observed over the past 2 decades, particularly among young women [1]. It comprises both suicidal and nonsuicidal behaviors and is predictive of poor social and emotional outcomes and future suicide attempts [2].

The positive and negative effects of interacting with web-based content on mental health, and especially on self-harm, are well documented [3,4]. The internet offers an important avenue for supporting people who self-harm, particularly those who are unable or reluctant to access formal, in-person support [5,6]. Increasingly, young people are likely to initially self-disclose self-harm behavior via the web owing to affordances of anonymity and perceptions of being judged less [7,8]. Young people experiencing suicidal feelings are also particularly likely to search for advice or peer support on the web [4,9,10]. However, web-based help seeking is also associated with risks, such as unintentional exposure to graphic or prosuicide and self-harm content and engagement with discussion forums in which self-harm is sometimes normalized and negative feelings are reinforced [3,8,9,11]. Therefore, it is vital that the web-based support content provided is safe, effective, and evidence-based.

Lived experience stories are a frequently used source of information and support on the websites of third-sector or health care organizations and can include user-generated content on social media [5,9]. Stories published by more formal help sites, such as Mind or Samaritans, typically involve a personal narrative of help seeking and recovery. Although limited research has focused on self-harm lived experience stories so far, there is emerging evidence suggesting that these narratives can inspire hope and help seeking in the reader [9]. A recent qualitative study compared *recovery narratives* presented live and recorded (in audio, video, or text-based formats) with adults using statutory mental health services in England [12]. Findings suggested that recorded narratives are valued for their availability *on demand*, for access to narrators without geographical constraint, and for the ability to access support without a need for social interaction [12]. In a mixed methods study including interviews with young people and general practitioners (GPs), Cohen et al [9] highlighted suicide-specific lived experience stories as a potentially useful and engaging source of web-based support. Simultaneously, concerns were raised by a GP that reading about someone else’s recovery could lead to negative comparisons and feelings of failure. A young participant suggested that detailed descriptions of the context in which an individual experienced suicidal ideation could be triggering to some readers.

### Objectives

Despite some evidence suggesting that creative storytelling [13] or digital storytelling [14] may be beneficial to oneself and others in terms of educating an audience and destigmatizing mental illness, little is known about how people who self-harm use web-based lived experience stories and how this may shape their behavior. With this in mind, we aimed to address the following research question—how do people who self-harm engage with, interpret, and evaluate lived experience stories accessed via the web?

## Methods

### Design

This was a qualitative focus group study.

### Recruitment

Participants for focus groups were recruited from another experimental study [15]. Interested participants were invited to complete an eligibility check and to provide their contact details. Participants were eligible to participate if they were aged ≥16 years, reported self-harm in the past year, and were fluent in English, with no other exclusion criteria. Purposive sampling—based on age and gender—was then used to invite a subset of those participants to participate in the focus groups. Written consent was obtained before each group session and confirmed verbally at the beginning of discussions.

Overall, 4 focus groups were conducted from June 2022 to July 2022, with a total of 13 participants in the United Kingdom. Owing to the sensitive nature of discussions, we limited the number of participants to 4 per group and attempted to assign participants to groups with others of similar age. This was not possible for gender, as only 2 men returned the consent forms. Groups consisted of 2 to 4 participants each.

### Procedure

Each focus group lasted between 40 and 60 minutes and was conducted via the web using Zoom (version 5.12.2; Zoom Video Communications, Inc) videoconferencing software. In total, 2 researchers were present to moderate each group. A semistructured approach was used. First, the lead moderator (LW) shared on the screen and read aloud 3 examples of lived experience stories about self-harm (Multimedia Appendix 1), followed by group discussion. The 3 example stories were created by combining extracts of *real-life* stories selected from different sources—including the websites of third-sector organizations, web-based forums, and personal blogs—to reflect a range of help-seeking circumstances. Each of the stories included a recovery narrative and did not include any graphic details of self-harm or encouragement of self-harm. Story 1 focused on self-help strategies and was written from the perspective of an individual aged 21 years. Story 2 did not explicitly report help seeking but conveyed a narrative of nonlinear recovery and a message of hope. Story 3 presented a journey of finding a therapist, medication, and social support. In the second and third stories, the age of the narrator was not specified. The example stories were used to prompt discussion, but participants were also encouraged to think about and discuss their own experience and reflect on other lived experience stories they had encountered on health care or third-sector organization websites or on social media.

Discussion was facilitated by flexible use of a topic guide [16] and included participants’ experiences of engaging with lived experience material on the web, their initial reactions to the example stories, specific aspects of stories (both in the examples and more broadly) that were supportive or not supportive, and the impact of reading lived experience stories on one’s own thoughts about help seeking and self-harm behavior. Participants received a shopping voucher worth £20 (US $24.64) by way of thanks, in addition to a debriefing sheet and signposting to support services. Discussions were audio-recorded and transcribed verbatim.

### Ethics Approval

The authors assert that all procedures contributing to this study comply with the ethical standards of the relevant national and institutional committees on human experimentation and with the Helsinki Declaration of 1975, as revised in 2008. All procedures involving human participants were approved by the School of Psychological Science research ethics committee at the University of Bristol (reference 10504).

### Analysis

Data were thematically analyzed, by following the 6 phases outlined by Braun and Clarke [17]. Anonymized transcripts were checked for accuracy and familiarization and then imported into NVivo (version 1.6.1; QSR International) for coding. LW and LB each openly coded the extracts from each of the 4 focus groups, collaboratively developing a coding frame to identify broad topics around which the codes clustered. Then, LW coded the full data set using the coding framework, added new codes, and iteratively refined the thematic descriptions and boundaries. Discussion with the study team led to further amendments to themes, with the transcripts then revisited to check accuracy. Our results are not presented separately for each story because the focus group discussions combined all 3 example stories and lived experience stories encountered elsewhere.

## Results

### Overview

Participants (11/13, 85% women and 2/13, 15% men) were aged between 16 and 40 years, and mean age was 24.5 (SD 7.3) years (Table 1).

Analysis generated 5 main themes related to how people with recent experience of self-harm interpret, evaluate, and engage with lived experience stories. These themes were labeled as follows: (1) stories of recovery from self-harm and their emotional impact, (2) impact on self-help and help-seeking behaviors, (3) identifying with the narrator, (4) authenticity, and (5) language and stereotyping.

In the descriptions of these themes in the following sections, we refer mainly to the discussions about specific features of the example stories presented to participants. Where participants referred to previous experiences of encountering stories on the web, these were most often accessed on mental health–related websites, such as Mind, but some participants also considered stories as user-generated content shared in web-based self-harm community forums or social media groups.

**Table 1:** Summary of characteristics of participants in each focus group (N=13).

Characteristics			Focus group 1 (n=2, 15%)		Focus group 2 (n=4, 31%)		Focus group 3 (n=4, 31%)		Focus group 4 (n=3, 23%)
**Gender, n (%)**									
	Men	0 (0)		0 (0)		1 (25)		1 (33)	
	Women	2 (100)		4 (100)		3 (75)		2 (67)	
**Age (years), mean (SD; range)**			24 (4.9; 20-27)		32 (8.1; 23-40)		18 (1.8; 16-20)		24 (1; 23-25)
	16-18, n (%)	0 (0)		0 (0)		2 (50)		0 (0)	
	19-21, n (%)	1 (50)		0 (0)		2 (50)		0 (0)	
	22-25, n (%)	0 (0)		1 (25)		0 (0)		3 (100)	
	≥26, n (%)	1 (50)		3 (75)		0 (0)		0 (0)	

### Stories of Recovery From Self-harm and Their Emotional Impact

Most participants described stories of recovery from self-harm—including positive framing of scars—as hopeful, motivating, or inspiring reminders of past struggles, helping to reassure them that they also could eventually recover in time. This included lived experience stories widely encountered on the web, with references made to specific features of the example stories:

Story one is just more look towards the future and kind of inspiring, maybe. Like that person did it so, so can I and everyone else...it’s definitely good to like know that once again, I am not the only one and it is reassuring and promising that other people can do it so, there is a chance for me too.

Woman; aged 19 years

Some spoke about general positive impact on their mood when reading the example stories with a positive outcome or motivational message that encourages patience and strength throughout a recovery journey:

...When they like kind of do their reflection at the end saying it is possible, I find that really does just pick me up a bit.

Woman; aged 20 years

...It just feels like the sort of thing, quotes that I’d like relate to or would like on Instagram. It’s quite nice little reminders, little pick me ups.

Woman; aged 24 years

However, older participants found it challenging if they encountered a story in any context that framed someone’s recovery within a certain period or by a certain age. The first example story was a recovery narrative from an individual aged 21 years. For participants aged >21 years, hearing about a young narrator’s recovery could be disheartening and could reinforce a sense of having failed to recover themselves:

There’s definitely an element of, you know, just the age thing again...if this is a teenage problem and I’m in my 20’s that means there’s no hope for me, that means I’m doomed, I’ll be like this forever.

Woman; aged 23 years

Some participants described the inclusion of numeric metrics in a lived experience story as being potentially problematic because it could encourage demoralizing comparisons. In discussions about the example stories presented, this commonly referred to the age of the narrator. Participants also reflected on the second example story and other stories they had encountered on social media, which included the number of days the narrator had not self-harmed:

Sometimes when people put the numbers or they’re like, “oh, I am so and so days clean” it’s quite hard because you feel like you are comparing all the time.

Woman; aged 20 years

### Impact on Self-help and Help-Seeking Behaviors

Several participants reported having actively sought out lived experience stories on organizational websites or on social media as a form of help seeking. This could be to lift their mood (refer to the *Stories of Recovery From Self-harm and Their Emotional Impact* section), as a means of distracting themselves from the immediate urge to self-harm, or to reduce feelings of social isolation:

It’s usually when I am feeling really low and that I don’t really know what to do with myself, you know, I don’t want to self-harm, I want to do something else. So, I try to see who else out there has gotten through it and just to see if there is hope out there because in those moments, there is literally nothing I would rather do than basically punish myself for whatever I am feeling...I think they just kind of calm me down enough to be able to actually do something that is healthier for me than self-harm.

Woman; aged 16 years

Although some participants reflected on the usefulness of reading lived experience stories on the web during urges to self-harm, others—reflecting on the example stories—discussed low mood as a barrier to the positive impact of a story. Several participants suggested that a reader’s frame of mind would influence their perception of a narrative or advice:

Once you have read this over and over and you think this is, in the immediate moment, the be all and end all of what is available to help you, it becomes a bit like, “fantastic, you can tell me the strategies – I’ve tried them, they’re not working. You can tell me that you’re supposed to have a support network – guess what, I don’t right now.”

Man; aged 25 years

A participant highlighted that, although she considered herself to be recovered, reading lived experience stories on social media was a cue that she should check on her own well-being and speak to her support network:

...If I read one of these and I am like, “Oh, actually I have been thinking about this a bit more” because sometimes the sign that you are reading one of these is a sign that actually, you’re thinking about it again.

Woman; aged 20 years

Reading lived experience stories—regardless of whether they were actively sought—was commonly reported to reduce feelings of isolation. This was often achieved simply by the recognition of others going through similar experiences or feelings. Some participants reflected on engagement with web-based communities to access user-generated stories, which also promoted a sense of social inclusion:

I’m just trying to find a place where I’m feeling less alone with these feelings.

Woman; aged 28 years

Practical ideas for self-help strategies were a key feature of the first example story and were described by most participants as particularly useful. Several participants reported encountering similar suggestions in stories read on organizational websites, and they had been inspired to try some suggested strategies in the past. Some participants suggested that further practical advice about where to go for help would also benefit readers:

I think I found the first story most helpful because of the suggestions of things that have helped. Things that you can try because it’s about thinking, actually what’s helped for someone else, that might help me. And having access to as many things as possible that might help because everything is different for everyone else...so that’s where I found that one most helpful.

Woman; aged 40 years

Highlighting the fundamental concept of self-help in the first example story was also described as motivating and important for those who do not have a support network or access to formal therapy. However, for some participants who reflected on content previously found on the web and the example stories, if the suggested strategies had been tried and tested or if the reader was feeling particularly low, advice could feel clichéd or irritating:

...Sometimes [friends who are trying to be supportive] will repeat the exact same phrases over and over and over. It just kind of makes you want to scream and I think stories like this can kind of have a similar effect if it’s something you’ve already seen a lot.

Man; aged 25 years

More broadly, lived experience stories on organizational websites could be sought to empower readers to better understand their own feelings and experiences:

I was more so actively seeking them perhaps when I wasn’t in treatment because I guess, it was a bit unknown or it was difficult to put pieces together. I guess that was more so perhaps with the trauma and sort of seeing how [self-harm] sort of is a coping mechanism for control or that other people [self-harm] for different reasons. So, I guess, last year, it was more when I was trying to figure things out maybe as a reassurance.

Woman; aged 27 years

Some participants described using lived experience stories previously accessed on organizational websites as a source of ideas for talking more confidently about their own experiences to others. This included speaking to their support network or therapist and sharing their own experiences to educate and help others:

I think I wanted to be able to talk about my own experience and found the best way to do that was to hear how other people talked about their own experience. Kind of, what do you include, what you don’t, what do people not include, those kind of things, how much detail do people go into, things like that.

Woman; aged 40 years

By learning about other people’s experiences and routes to help seeking, participants appeared to recognize a need for help in others, and in turn, recognized their own need to seek support. This could be through learning about others’ routes to therapy. More frequently, participants described the knowledge that they were not alone being a catalyst for help seeking when they had encountered lived experience stories in the past. The following quote refers specifically to the example stories presented to participants:

It’s hard to connect to just like words on a screen but I think these stories do a pretty good job at that and I think it will help a lot of people to then seek help because they realise, “I am not alone. I am not crazy, and I deserve help for this, and I need help.”

Man; aged 17 years

### Identifying With the Narrator

All participants reported identifying with at least one aspect of ≥1 example lived experience stories. This type of web-based content was suggested to be intrinsically more relatable than advice from a health care professional:

You can have a therapist or a professional telling you do X and Y but I found for me, I didn’t really listen to them as much as I would another person who may or may not have been a similar age but has lived through a similar thing as me...Hearing it from someone who has gone through it is so much more impactful.

Woman; aged 20 years

Most participants suggested that it was more important to be able to relate to a narrator’s feelings or experiences within a lived experience story than to their demographic background. By keeping the narrator’s age and gender ambiguous in the example stories (the age was specified in the first example story), older readers, in particular, seemed to be able to project their own characteristics onto the story, thus making it feel more relevant and engaging to them. However, some young participants found it easier to relate to stories from similarly aged narrators, which brought them “comfort” (man; aged 17 years).

Some participants felt that organizational websites should provide several stories that reflect a range of experiences to encourage readers to identify and connect with them. However, others noted that being very specific about the circumstances preceding a narrator’s self-harm—or about co-occurring mental health disorders or methods of self-harm—could alienate some readers by invalidating their feelings:

I don’t find it helpful when people give like loads of reasons, like this bad thing happened, and then I self-harmed. Because I found – I don’t come across that so much now but when I was younger, I found that made me think I don’t deserve to self-harm, I’ve not had those experiences. And it just felt like another way of invalidating me, but there’s not a huge amount of that in these [example] stories...It’s not “oh look at me and all the horrible things I’ve been through.”

Woman; aged 38 years

Several participants noted the challenge of organizational websites needing to provide stories from multiple perspectives to appeal to the broadest audience, acknowledging that an exhaustive collection would be impossible:

...If it’s too generic and vague, it’s almost like it’s not real, but if you make it too specific then obviously you narrow down the people who can perhaps relate to it. But then who’s going to want to sit and read through ten different accounts [stories] to find one that matches their own experience?

Woman; aged 38 years

Identifying with the narrator also appeared to be an important factor in encouraging help seeking through the recognition of need in others and thus oneself (refer to the *Impact on Self-help and Help-Seeking Behaviors* section) but was simultaneously reported by most participants as unlikely to affect the likelihood of future self-harm behavior, as each individual’s self-harm experience is unique:

For me, these stories don’t really affect what I would be doing because obviously, it is someone else’s experience and not mine. So, I feel like it wouldn’t impact me as much and what I do in the future.

Woman; aged 19 years

### Authenticity

The example lived experience stories, which were moderated to avoid being triggering, were reported by some participants as feeling sanitized and somewhat unrealistic. Although some participants suggested that more “uncomfortable” stories with more extreme accounts of crisis would be more likely to prompt them to seek help, others recognized the risk that this could be triggering to some readers:

I think these stories are kind of sanitised versions of the actual experience. So I don’t think for them there’s really anything that could be particularly triggering. I’m kind of in two minds in terms of a more extreme story being helpful. But I think some people it definitely can be because it kind of makes you face up to the potential thing that’s going to happen. I also think that could cause different people to spiral a little bit thinking, “oh God, this is going to happen to me,” not “this could happen to me.” But I think that is very much on an individual basis, I’m not trying to say that story would be bad or that story would be good, it’s just it would potentially be a difficult one to kind of balance the benefits with the potential risks for some.

Man; aged 25 years

Some aspects of the example stories were felt to increase a sense of authenticity and "humanize" the story without the need for the storyline to be extreme or graphic. First, having realistic representations about recovery being a nonlinear process was considered to be helpful in terms of managing expectations and pressure on participants for their own recovery. The second example story reflected the likelihood of setbacks or relapses during the process of recovery. This was interpreted by several participants as more authentic and particularly relatable (refer to the *Identifying With the Narrator* section):

I liked this one perhaps not so much because of the self-care ideas or things but because it shows sort of the ups and downs of the trajectory and I think that sort of humanizes it a bit and I think that’s important. I think accepting that is probably one of the most important things, and accepting that it’s not an upwards linear trajectory, it’s up, down, down, up, so, I found that one quite helpful because of that.

Woman; aged 27 years

Second, acknowledgment of the challenges attached to accessing therapy (in the third example story) was considered by some participants to be helpful in setting realistic expectations. However, others noted that excessive focus on the difficulties around therapy could be discouraging:

It’s tricky, but from my point of view, it’s about trying to find that middle ground between saying “I went to therapy and everything’s good now because therapy’s amazing” and saying “I’ve been to 97 therapists and 96 of them were dreadful and it’s taken 30 years” or whatever and then you think, “oh well there’s no point then.” Because obviously everyone’s experience is going to be different, so I think it’s trying to find a middle ground without being too scripted.

Woman; aged 23 years

Third, references to scars (in the third example story) were not reported by any participant to be triggering but served to make a story feel more real and “one of the more lived parts of that lived experience” (man; aged 25 years). The positive framing of scars in this example story was felt by several participants as contributing to a general uplifting and motivating effect. A participant further noted that including a reference to scars could be an important deterrent of self-harm, by reminding the reader about its lasting effects:

If anything that’s probably like one of the lines. I mean it wouldn’t stop me from doing it, but if any of the lines were that would probably be the most likely to stop me from self-harming; to know that there are lasting effects, and actually it does leave scars. Because I think it’s hard to remember when you’re doing it at the time that you’re going to be reminded of it for the rest of your life.

Woman; aged 24 years

Finally, in the second example story, the narrator reflected on the importance of their self-harm as a means of coping and as part of their identity. This seemed to particularly appeal to and be respected by participants. Acknowledgment of self-harm as a coping mechanism appeared to reduce feelings of stigma and contributed to increased understanding of participants’ own experiences:

At the end of the day you’ve sometimes got to accept that in the relative scheme of things, that might be the healthiest coping mechanism you’ve got at hand.

Man; aged 25 years

Although this was presented positively by some participants as feeling authentic, it is also important to note the potential negative impact this normalization of self-harm may have on help-seeking behavior.

Several participants reflected on the authentic representation of self-harm as an important part of the narrator’s personal or social identity in the second example story. Some participants linked this to engaging with lived experience stories on social media and interacting with other members of web-based self-harm discussion boards or groups. Reflections on identity and engagement with web-based groups were felt to be helpful in providing a sense of social support by knowing that there were others going through similar experiences. However, the same participants simultaneously felt this sense of identity could hinder someone’s recovery if stopping self-harm was accompanied by detachment from the self-harm community:

Yeah, I think it’s like we’re all part of this kind of secret community that nobody knows about unless you’re part of it. And then if something happens and you leave the community then you’re not part of it anymore. It feels like [someone else] said about having [self-harm] as part of your identity and this is how I am, then it’s not a part of who you are anymore it’s almost like you’re losing that, but then you’re losing that community as well.

Woman; aged 28 years

This was felt most acutely when referring to engagement with lived experience stories as user-generated content on social media and web-based forums, which could sometimes feel like an unhealthy, competitive space in which self-harm was normalized:

I think although it’s reassuring to know there’s other people like you, it can also be a bit of a toxic environment because everybody is talking about the same thing and it’s almost like normalizing it. And I suppose although it shouldn’t be something you’re ashamed of, you know, it almost is encouraging this behavior sometimes, I think. And can be – it can kind of lull you into a false sense of security I think in these online spaces particularly. And it can kind of feed that negative mindset of like, okay I’m doing this but if I don’t want to stop doing it then this is the place I’ll go.

Woman; aged 28 years

### Language and Stereotyping

Several participants reflected on the importance of lived experience stories using language that does not stigmatize self-harm or alienate people who engage in self-harm. When lived experience stories referred to self-harm as an unhealthy coping mechanism—discussed hypothetically and in reference to the second example story—some participants felt that it reinforced a sense of moralistic judgment, which could feel devaluing. This also applied to the use of the word, “clean,” in the second example story, when the narrator described the number of days they had not self-harmed. Although some participants reported feeling ambivalent about this terminology, others highlighted the stigmatizing effect this may have on people who self-harm. A participant discussed the potential harm this stigma presents in deterring people from seeking support:

I didn’t think of the comparison to drugs first, but comparison to STD’s and in particular HIV and the stigma around that. And I think all three of them kind of interplay in this idea that it’s a moral judgement on a medical issue. It isn’t helpful to put that moralistic judgement on something like that, because it isn’t helpful to people either for feeling able to seek help or finding help in a safe way. And then it doesn’t make it as accessible because it isn’t as easy to talk about. So I kind of particularly think that [being “clean”] is questionable wording in that respect. I think also by using it to give it that moralistic judgement, people who are going through that are more likely to feel worse in themselves.

Man; aged 25 years

A participant highlighted the term, “cutting,” in the third example story—but also encountered as “cutter” in other stories found on the web—as “derogatory” and “crass” (woman; aged 28 years). This sort of terminology was also felt to be problematic for being overly specific and thus potentially alienating some readers (refer to the *Identifying With the Narrator* section):

I suppose also there’s other ways of self-harm and it’s not just cutting so that – by saying that it almost invalidates saying “oh it’s not self-harm because it’s head banging, or it’s not self-harm because it’s overdoses” or something.

Woman; aged 40 years

The example stories and those previously encountered on organizational websites were sometimes felt to perpetuate the stereotype of self-harm as a problem for teenage girls (refer to the *Identifying With the Narrator* section). Several participants suggested that providing a wide range of stories on organizational websites, including some from older narrators, would be beneficial:

They’re quite young in all the stories. I think it would be nice every now and then to see a story of like somebody a bit older. Because I feel like a lot of self-harm is sort of people assume it’s always like teenagers who are depressed or something. But it would be nice if there was more like 20, 30-year-old stories. So, you know that actually it’s not just depressed teenagers that do it.

Woman; aged 24 years

## Discussion

### Principal Findings

People with recent self-harm experience reported positive engagement with lived experience stories encountered on organizational websites and sometimes on social media. Example stories used to evaluate different features of this type of web-based content were generally found to be most engaging and helpful if the feelings recounted by the narrator were relatable, practical help seeking and self-help strategies were included, the stories felt human and authentic, and the language used did not perpetuate stigma or stereotypes about self-harm. Our findings suggest that lived experience stories can support readers to feel validated and help them to better understand their own feelings and experiences. Participants also told us that exposure to these stories can empower people to share their experiences with professionals and their support network and that they may also provide a helpful way of educating others about self-harm, which can reduce stigma and self-stigma [18].

The narratives of recovery were generally felt to be inspiring and motivating to those thinking about their own recovery from self-harm, and lived experience stories appeared to be highly valued as a form of web-based support, which could also serve as a temporary distraction for those experiencing an immediate urge to self-harm. However, our findings also highlight that voicing such narratives from a young or emerging adult’s perspective could have a demoralizing effect on older readers. Strategies to mitigate this problem may include (1) maintaining ambiguity regarding the age of the narrator to minimize exclusion of some readers; (2) including more stories from older narrators to broaden the representation of different age groups; or (3) tailoring age references depending on the target audience, for example, using young narrators on youth mental health websites.

Our findings also highlight the need for a balance between providing detail and specificity to make stories feel relatable to readers and avoiding the exclusion or invalidation of readers who do not identify with such details. Previous studies suggest that high specificity in stories about self-harm or suicide—particularly regarding methods—can risk overidentification with the individual or narrator, thus increasing the risk of contagion [7,19,20]. This was not captured in our findings as we deliberately excluded detailed references to methods of harm for ethical reasons. A balance should also be sought between the avoidance of narratives that may be triggering for some readers and the use of stories that could be overly formulaic and sanitized. In our sample, including a positive reference to scars in an example story was not felt to be triggering but contributed a sense of the lived experience story being more raw and real. Authenticity was felt to be further enhanced through portraying recovery journeys as nonlinear and acknowledging relapses and thus managing expectations regarding one’s own recovery. Although care must be taken to avoid normalization of self-harm, acknowledgment of self-harm as a key coping mechanism or part of someone’s identity may also feel less sanitized to some readers, thus increasing their engagement with a story.

Our findings highlight lived experience stories as a setting for the reader to compare themselves with the narrator. This can be helpful in managing feelings of isolation, knowing that others are experiencing similar feelings, and engaging in self-harm as a coping mechanism. It can also be positive when viewing stories of recovery as motivating. Feeling as if one is in a position comparable with that of the narrator may inspire help seeking by recognizing need in others and oneself by extension and by instilling hope that recovery can be attained. However, not all comparisons are positive. We found that the helpfulness of comparisons between reader and narrator may depend on their relative age and the reader’s stage of recovery. Having few negative experiences or those perceived to be less traumatic compared with those of the narrator can feel invalidating and may deter some from seeking help as a result. Comparing oneself against a narrator who is more advanced in their recovery may instill feelings of dejection and pessimism in the reader. Providing numeric metrics, such as days without self-harm, can make comparisons particularly salient. Although comparing with others going through similar experiences can reduce feelings of isolation and encourage a sense of community, web-based forums can also foster a sense of toxic competition and normalization of self-harm.

Feelings of connectedness, enhanced understanding of one’s own emotions and behavior, use of alternative coping strategies for emotion regulation, and self-disclosure of self-harm to others have been described elsewhere as important factors in recovery from self-harm [21]. Thus, our findings suggest that engagement with web-based lived experience stories may provide a worthy contribution to the self-harm recovery process.

### Limitations

It is important to recognize that the example stories presented to participants in this study were selected by the research team to be recovery oriented and consistent with safety guidelines regarding web content and thus excluded graphic content. This is reflected in some of the comments about stories feeling *sanitized* (refer to the *Authenticity* section). Although these stories were felt to be broadly representative of content published by third-sector organizations, they may not reflect unmoderated, user-generated content shared on social media. Participants’ accounts of encountering uncensored or less-helpful stories were limited. Therefore, our findings highlight several helpful aspects of lived experience stories, but we are limited in the extent to which we can draw conclusions about the potentially harmful aspects. This is particularly relevant to stories shared on social media, which may include more graphic content.

Similarly, although our focus was on specific features of the example stories and those encountered on organizational websites, there was limited discussion about user-generated content encountered on the web. Therefore, we have not explored the potential impact of purposeful reading of a lived experience story—one that is expected and consented to—compared with unexpectedly encountering a story on social media by its appearance on a feed or by algorithmic promotion.

We purposely limited the numbers in each focus group owing to the sensitive nature of the topic; however, group 1 was smaller than intended. Although the 2 participants in group 1 appeared to be comfortable with reflecting on personal and upsetting experiences, the discussion flowed more freely in the other groups, each with 3 or 4 participants. Participants in the slightly larger groups were better able to respond to one another, and concurrence and disagreement among group members resulted in particularly rich data.

Our sample was not diverse in terms of gender, with boys and men being particularly underrepresented. This is a common challenge in self-harm studies [5], but our additional attempts to purposively sample this group were unsuccessful. Information about participants’ ethnicity, sexual orientation, socioeconomic status, geographical location, or whether they were transgender individuals was not collected, making it impossible to evaluate the diversity of the sample. However, a broad age range was represented in our sample, thus providing valuable insight into the age-related factors discussed previously. In addition, many participants described being at different stages of a recovery journey, thus contributing useful insight into differences in acceptance, reactions to, and engagement with lived experience stories, depending on the current circumstances or mindset of the reader.

### Comparison With Previous Studies

There has been little previous research on engagement with lived experience stories related to self-harm. Interviews with young people and GPs in the United Kingdom about web-based support services for suicide prevention indicated the overall value placed on lived experience content [9]. Consistent with our findings, young people reported the feeling of reassurance that they were not alone in their experiences, recognition of the need for support in others and themselves in turn, and concerns around content being potentially triggering to vulnerable readers. The potential for negative comparison was also raised by GPs, which we highlight as a particular risk for adults who self-harm beyond adolescence. We further emphasize that the inclusion of numeric metrics (such as age and days free from self-harm) in a story may increase the likelihood of comparison.

People who engage in web-based help seeking for self-harm have described stumbling across graphic social media content or comments that reinforce self-harm and suicide [6,19]. Our findings suggest several positive aspects of lived experience stories similar to those provided on organizational websites (such as Mind or Samaritans). Although we did not focus in depth on graphic content within lived experience stories on social media, our findings indicate that there may be risks of competition attached to engagement with user-generated content, which is often accompanied by unmoderated interactions [5]. Such feelings of competitiveness have previously been identified as a negative outcome of (and motivation for) engagement in web-based self-harm forums [6,7].

Our findings align with the framework for impact and moderators of impact developed by Rennick-Egglestone et al [22] in a systematic review of recovery narratives related to eating disorder and psychosis. By synthesizing 4 qualitative studies on the impact of recovery narratives (the key thread of most lived experience stories), the authors identified 6 themes, all of which were reinforced by our own findings specific to self-harm. The themes identified in the review by Rennick-Egglestone et al [22] were *connectedness to the narrator or to others*; *understanding of mental illness and how to recover*; *reduction of stigma, including self-stigma*; *validation of self and of personal experiences*; *affective responses*; and *behavioral responses*. Several moderators of impact were also identified regarding reader and narrator characteristics and are reflected in our findings; however, contextual characteristics (such as internet access) were not relevant in our study. Consistent with our findings, positive reception of a story was facilitated by the reader having similar life experiences to the narrator, whereas barriers included inability to relate to the narrator’s recovery journey or feeling worse than the narrator. As evidenced in our findings, Rennick-Egglestone et al [22] suggest the perceived authenticity of the narrator to be a key moderator of the impact of a narrative. This review was part of an in-depth body of work by the Narrative Experiences Online team to evaluate the impact of recovery narratives on adults who experience psychosis or other mental health problems [23]. In addition, within the Narrative Experiences Online program, Ng et al [20] have developed a causal chain model, using data from interviews with adult users of UK mental health services, to demonstrate the key mechanisms in whether a reader connects with a recovery narrative. These were (1) comparison with the narrator, in terms of shared experiences, stage of recovery, and narrator characteristics; (2) learning, in terms of gaining insight into the perspectives of others and developing new coping techniques; and (3) empathy, in terms of insight into the struggles, successes, and tone of the narrator. Our findings provide further support for these mechanisms of connection, extended to the self-harm context. We additionally highlight empowerment through enhanced understanding of one’s own experience as a key part of the *learning* mechanism and both avoidance of stigmatizing language and authentic representation of a self-harm recovery journey as key parts of *empathy*.

However, in contrast to the findings by Ng et al [20], and perhaps specific to the self-harm context, we found that lived experience stories were felt to be a useful distraction and source of support during moments of crisis for people who self-harm. Participants in our study also interpreted nonlinear narratives of recovery as more authentic and easy to connect with, whereas disjointed or circular narratives were interpreted pessimistically by Ng et al [20]. Nonlinear narratives of recovery from self-harm may be useful if they project an overall upward trajectory.

As mentioned in the *Limitations* section, the example stories presented to participants were carefully selected and were more representative of stories published on organizational websites than on social media, in terms of more *sanitized* content and a clear narrative structure. Although our findings point to several suggestions for helpful aspects to be included in a lived experience story, people posting a story on the web or those moderating web-based forums should also be mindful of existing guidelines around safe communication about suicide and self-harm on social media [6,24,25]. Broadly consistent with our findings (refer to the theme, *Language and Stereotyping*), #chatsafe guidelines recommend removing unhelpful language or graphic references to self-harm and using trigger warnings if appropriate [24].

### Conclusions

This study highlights the value placed on lived experience stories as an accessible form of web-based support and information for those who self-harm. Our findings have several implications for health care or third-sector organizations publishing this type of content and for community guidelines for web-based forums in which users share their stories. First, it is important to consider the narrator’s age when describing a successful recovery. Second, ensuring that stories feel human and relatable by including references to challenges and relapses in a recovery journey will be valuable. Third, care should be taken to not perpetuate stereotypes of self-harm as a problem for teenage girls, and stigmatizing language or implied moral judgments should be avoided. We urge caution to those publishing or moderating lived experience stories, in terms of removing unhelpful language or graphic references to self-harm, using trigger warnings if appropriate, and being mindful of the risk of negative comparisons or competition.

Lived experience stories—shared carefully—have the potential to inspire those who self-harm to seek help, empower people to understand and share their own experiences, distract those experiencing an urge to self-harm in the moment, and reduce feelings of self-stigma and isolation.

## Appendix

Multimedia Appendix 1 The 3 example lived experience stories presented to focus group participants for discussion.

## Abbreviations

GP: general practitioner
