# Supplementary material for: Investigating How People Who Self-harm Evaluate Web-Based Lived Experience Stories: Focus Group Study
Source: JMIR Ment Health. 2023 Jan 31;10:e43840. doi: 10.2196/43840 (PMC9929729; doi:10.2196/43840)
Supplement: Multimedia Appendix 1 [file mental_v10i1e43840_app1.docx]

# Supplementary material for: How do people who self-harm evaluate online lived experience stories? A focus group study

## **Example lived experience stories presented to focus group participants**

**Story 1**

I started self-harming when I was 15. I was bullied really badly, and I was so lonely. I didn’t understand why I was getting picked on. I thought it had to be me that’s the problem, and that’s when I started to self-harm and think about suicide.

I carried so much anger with me all of the time. When I lost all my friends, that anger had to go somewhere. It seemed like the easiest thing was to inflict pain on myself. I saw it as a punishment. I thought I was such a bad person.

Nowadays, to manage my emotions, I read a lot: I always have self-care books on my phone. If I feel anxious, it’s about physically bringing myself back into the room. I smell something, taste something and touch something. I do breathing exercises, and once I feel like I’m ok I put on something to do with self-help, on iTunes, Amazon or YouTube: something that is gentle but is inspiring.

I read a lot about mindfulness, accepting where you are in life. I’ve found that when you accept the here and now, life will become a lot easier. That’s incredibly powerful for me.

When I was self-harming, if someone had said to me when you are 21, you are going to be happy and healthy, I wouldn’t have believed them.

But here I am.

**Story 2**

I don’t think I realised that what I was doing was self-harming until I was about 16 (I started at 14). I had figured it out as a way to hurt/punish myself and I kept it secret for a long time.

There were lots of relapses but because I was putting the effort in and working on myself, I was using healthy coping techniques instead and things seemed to improve overall. Relapses can be disheartening because you can feel like you’ve made so much progress by being say 40 days clean and then you break it and it can feel like you’re back to square one. But instead of pressuring myself to be 41 days clean the next time I would just focus on staying clean for however long I could.

Be easy on yourself because recovery is not linear. It’s forwards, backwards, up, down and round and round but when you commit to getting yourself to a better headspace and remember that self-harm is not the answer to all your problems, things will get better. The setbacks shouldn’t matter as much, it’s how you pick yourself back up again.

It's totally okay to miss it and grieve for the loss of something that was a big part of who you were at one point in time. That's totally fine. And again it doesn't mean it has to be a thing you keep doing. You can feel those things without having to act on the urges.

Finally, it is possible. I didn’t think I‘d be able to do it in a million years but it does get easier over time. Be patient, stay strong and you will get there, I promise.

**Story 3**

Around 2010/2011, I was getting very stressed about life in general. My parents were pushing me to find a job, among other things. Over the course of a few months, it got to the point where I was starting to feel like I wasn’t living up to their expectations, and that I was worthless. At that point, I started feeling numb, and I started cutting. I had been feeling emotionally numb at the time, and that I was losing control of my life. Cutting gave me a sense of control over something even as I felt like my life was falling apart around me.

My mom was the first one to really notice how my behaviour changed, and along with it my personality. I wasn’t really interested in the things I had been interested in, I was eating and sleeping more, and was pretty much moping around the house. Every time she would ask me something, I would answer with “I don’t know” or “probably not” about 90% of the time.

It took me a couple of months before I realised that I needed help. My mom and I went to see a few therapists, but the first two I saw I didn’t really connect with. The third therapist I pretty much immediately clicked with. During the initial meeting I was told I had clinical depression. I was put on medication, and I continued to see my therapist throughout the whole ordeal, which helped.

Initially not much seemed to change. I still had thoughts of cutting when things got really stressful. After a few months, though, I started noticing the changes. Then, I started dating my current boyfriend. Early on in the relationship, I did tell him that I used to cut and that I had been diagnosed with depression. Whenever I had a bad episode or got really upset, I would either text or call him and he would talk to me to help me think of something else other than cutting. That definitely helped.

Now I am off the medication, and have not thought about cutting for the last 7 years. I have no plans on getting rid of the scars from then, as I see them as a reminder of how I had hit a low point in my life and survived.
